# Measurement of serum C-reactive protein concentration for discriminating between suppurative arthritis and osteoarthritis in dogs

**DOI:** 10.1186/s12917-016-0868-4

**Published:** 2016-10-28

**Authors:** Anna Hillström, Jonas Bylin, Ragnvi Hagman, Karin Björhall, Harold Tvedten, Kristian Königsson, Tove Fall, Mads Kjelgaard-Hansen

**Affiliations:** 1 Department of Clinical Sciences, Swedish University of Agricultural Sciences, Uppsala, Sweden; 2 Evidensia Södra Djursjukhuset, Stockholm, Sweden; 3 Department of Translational Sciences, RIA IMed, AstraZeneca R&D Mölndal, Mölndal, Sweden; 4 Safety Assessment, AstraZeneca R&D Södertälje, Södertälje, Sweden; 5 Department of Medical Sciences, Molecular Epidemiology and Science for Life Laboratory, Uppsala University, Uppsala, Sweden; 6 Translational Haemophilia Pharmacology, Novo Nordisk, Maaloev, Denmark

**Keywords:** C-reactive protein, Immune-mediated arthritis, Interleukin 6, Osteoarthritis, Septic arthritis, Suppurative arthritis, Synovial fluid

## Abstract

**Background:**

In a dog with joint pain, it is important to determine whether it has suppurative joint disease, characterized by exudation of neutrophils in the synovial fluid, or not, as this affects choice of diagnostic tests and treatments. The aim of this study was to evaluate whether measurement of serum C-reactive protein (CRP) concentration could be used to discriminate between dogs with suppurative arthritis and osteoarthritis (OA). Furthermore, the concentrations of serum and synovial fluid interleukin (IL) 6 concentrations were measured in dogs with joint disease and in healthy dogs, and were correlated to serum CRP concentrations.

**Methods:**

Dogs with joint pain were enrolled prospectively and were classified to have suppurative arthritis or OA based on synovial fluid analysis and radiographic/arthroscopic findings. Healthy Beagles were enrolled as a comparative group. CRP and IL-6 concentrations were measured with canine-specific immunoassays. The performance of CRP concentration in discriminating between dogs with suppurative arthritis and OA was evaluated using a previously established clinical decision limit for CRP (20 mg/l), and by receiver operator characteristic (ROC) curve and logistic regression analysis. Comparisons of CRP and IL-6 concentrations between groups were performed using t-tests, and correlations by Spearman rank correlation coefficients.

**Results:**

Samples were obtained from 31 dogs with suppurative arthritis, 34 dogs with OA, and 17 healthy dogs. Sixty-two out of 65 dogs with joint disease were correctly classified using the clinical decision limit for CRP. Evaluation of ROC curve and regression analysis indicated that serum CRP concentrations could discriminate between suppurative arthritis and OA. Dogs with suppurative arthritis had higher serum CRP and serum and synovial fluid IL-6 concentrations compared to dogs with OA (*p* < 0.001). Dogs with OA had higher synovial fluid IL-6 concentrations (*p* < 0.001), but not higher serum CRP (*p* = 0.29) or serum IL-6 (*p* = 0.07) concentrations, compared to healthy dogs. There was a positive correlation between synovial fluid IL-6 and serum CRP concentrations (r_s_ = 0.733, *p* < 0.001), and between serum IL-6 and serum CRP concentrations (r_s_ = 0.729, *p* < 0.001).

**Conclusion:**

CRP concentration was found to discriminate well between dogs with suppurative arthritis and OA.

## Background

Osteoarthritis (OA), septic arthritis and immune-mediated arthritis are important causes of joint pain in dogs. Osteoarthritis is a slowly progressive condition with deterioration of articular cartilage, osteophyte formation, and changes in periarticular tissues [1]. Although historically considered to be a non-inflammatory disease, there is evidence that local inflammation plays a part in the pathogenesis of OA [2], and increased activity of pro-inflammatory cytokines have been found in synovial fluid (SF) of affected dogs [3, 4]. However, neutrophil numbers in the SF are not increased in dogs with OA [5]. This is in contrast to dogs with septic and immune-mediated arthritis, which both are characterized by suppurative arthritis with exudation of neutrophils in the synovial fluid [6–8].

When a dog is admitted to veterinary care because of joint pain, it is important to rapidly determine whether suppurative arthritis is likely or not, as this affects the choice of further diagnostic testing and treatments. In a dog with suppurative arthritis, SF analysis should promptly be performed and treatment with antibiotics or glucocorticoids is often needed [7, 9]. This is in contrast to OA, which is usually a less acute condition not requiring arthrocentesis for SF analysis. Although findings at physical examination often differ in the two conditions, with signs of systemic inflammation frequently being present in dogs with suppurative arthritis but not in OA [1, 7, 8], the clinical picture is not always clear [8, 10, 11]. A circulating biomarker that could discriminate between suppurative arthritis and OA is thus wanted. In cases of suppurative arthritis, it is further necessary to establish an etiologic diagnosis as treatment strategies are dependent on the cause of inflammation [6, 7]. The differentiation of septic and immune-mediated arthritis can be challenging because results from SF analysis are similar, and specific findings such as bacteria, ragocytes and lupus erythematosus cells are often absent [5]. Moreover, the result from SF bacterial culture is not rapidly available, and false negative culture results are common [6, 12]. Hence, there is need for alternative biomarkers with the potential to discriminate septic from immune-mediated arthritis.

C-reactive protein (CRP) is an acute phase protein produced in the liver in response to increased concentrations of interleukin (IL) 6 during inflammation [13, 14]. Measurement of circulating concentrations of CRP in dogs has been shown to be clinically useful for diagnosing and monitoring systemic inflammatory diseases [15, 16]. C-reactive protein has the advantages of being an objective, quantitative marker of inflammation [17] that is not biased by treatment with non-steroidal anti-inflammatory drugs (NSAID) [18–20] or glucocorticoids [21]. This is in contrast to other markers of systemic inflammation such as fever and evaluation of the leukogram [21, 22]. Because serum CRP concentrations have been reported to be substantially elevated in suppurative arthritis [23, 24], but not in OA [25, 26], our hypothesis was that this marker should help differentiate between these two conditions. The aim of this study was to investigate whether measurement of serum CRP concentration could discriminate between dogs with suppurative arthritis and dogs with OA, and between dogs with septic suppurative and immune-mediated suppurative arthritis. An additional aim was to measure and compare concentrations of serum CRP, serum IL-6, and SF IL-6 in dogs with suppurative arthritis, OA, and in healthy dogs, and to investigate the correlation between IL-6 and CRP concentrations. The current study, comprising dogs with various degrees of joint inflammation, constituted a suitable setting for gaining additional knowledge about IL-6 and CRP concentrations in dogs with naturally acquired inflammatory disease.

## Methods

### Animals and samples

Client-owned dogs were enrolled to the study prospectively from the University Animal Hospital, Swedish University of Agricultural Sciences (SLU), Uppsala, or Evidensia Södra Djursjukhuset, Stockholm, from January 1^st^ 2012 to December 31^st^ 2013.

Inclusion criterion was at least one painful joint as determined by a veterinarian at physical examination. Furthermore, it was required that the dog should have arthrocentesis, arthroscopy or arthrotomy performed for diagnostic or therapeutic purposes, and that one of the authors (AH or JB) were attending during the procedure. Exclusion criteria were pregnancy and glucocorticoid treatment within four weeks prior to sampling. The veterinary surgeon in charge was responsible for the management of the dog, which was not affected by participation in the study. Medical records were studied for identifying number of painful joints, presence of other disease, treatments, and for follow-up during a period of at least 2 weeks after sampling. Blood samples were collected from the distal cephalic vein in EDTA tubes and tubes without anti-coagulant^1^. Samples were centrifuged after 30 min (5 min, 3000 G) and sera immediately transferred to cryo-tubes^2^ and frozen at −80 °C, or placed in liquid nitrogen until transferred to −80 °C. Synovial fluid samples were aseptically collected by arthrocentesis from 1–4 painful joints of each dog, prior to any other interventions. The number of joints sampled was determined by the veterinary surgeon in charge. For cytokine analysis, one aliquot of SF was centrifuged (5 min, 450 G) within 15 min and the supernatant transferred to cryotubes and immediately stored at −80 °C, or placed in liquid nitrogen until transferred to −80 °C. The remaining SF was used for nucleated cell count (NCC), cytological examination, and bacterial culture.

Eighteen purpose bred Beagle dogs, euthanized for reasons other than participation in the present study, were sampled at AstraZeneca R&D, Södertälje, and SLU. Inclusion criteria for the control dogs were physical examination without abnormal findings, no history of medical treatment during the past two months, and that the caretaker reported the dog to be healthy. Blood samples were collected from the jugular or distal cephalic vein immediately prior to the dog being euthanized. Synovial fluid samples were obtained while the dog was under general anaesthesia, or within 5 min after euthanasia. Dogs with macroscopic joint lesions at post-mortem inspection, or abnormalities on laboratory tests results from blood and SF, were excluded. Samples from the healthy dogs were handled similarly as described above for the samples from cases. Maximal storage time for all serum and SF samples was 2 years.

### Analyses

#### Hematology, biochemistry and serology

A routine biochemistry profile was performed on an automated analyser^3^ in all of the dogs. The complete blood cell count was assessed with an automated hematology analyser^4^^,^ 5 within 6 h after collection, including manual white blood cell (WBC) differential count. Results from hematology and biochemistry analyses were compared to locally established reference intervals for adult dogs. Sera were analysed for the presence of antibodies against *Borrelia burgdorferi* and *Anaplasma phagocytophilum* with an indirect immunofluorescent-antibody assay at the National Veterinary Institute, Uppsala, Sweden [27].

#### Synovial fluid analyses

Microscopic examination of SF including a nucleated cell differential count was performed by a clinical pathologist (AH), who was blinded for history and clinical findings at the time of examination. The SF nucleated cell count (NCC) was assessed to be normal if ≤3 cells per high-power field (100 x oil-immersion objective) were found [5], with ≤5 % neutrophils. The presence of mild, moderate or marked increased NCC on cytological examination was subjectively assessed, as well as erythrocyte numbers. The smears were screened for the presence of microorganisms, ragocytes, and lupus erythematosus cells [5]. When SF was available from multiple joints from the same dog, each joint was evaluated separately. Pathological bleeding was diagnosed if macrophages containing erythrocytes and/or hemoglobin breakdown pigment were found [5]. Iatrogenic bleeding was defined as presence of moderately to marked numbers of erythrocytes, without signs of pathological bleeding. When SF volume was sufficient, nucleated cells were counted using a hemocytometer (BÜrker chamber) after treatment with 0.5 mg/ml hyaluronidase^6^ at 37 °C for 30 min. Bacterial culture was performed by adding >1 ml of SF to aerobic medium blood culture containers^7^, that were cultured at 37 °C for maximum 7 days or until growth at the Section of Bacteriology, National Veterinary Institute, Uppsala, Sweden. In cases where the SF volume was not sufficient for culture in blood culture containers, SF was instead transferred to a sterile cotton swab^8^ that was placed in enrichment media prior to culture on horse blood and bromcresole lactose purple agar plates.

#### Analysis of serum C-reactive protein and serum and synovial fluid IL-6

Serum CRP concentration was determined with a previously validated canine-specific CRP assay^9^ with a measurement range of 6.8-300 mg/l [28], on a fully automated, open-system clinical chemistry/immunoassay analyser^c^. Samples were analysed in duplicate in random order in a single run. Samples with serum CRP concentrations <6.8 mg/l, which was the limit of quantification (LOQ) of the CRP assay, were immediately re-analyzed in duplicate with a validated automated high-sensitivity CRP test^10^ with a LOQ of 0.5 mg/l [29]. Measurement of IL-6 was performed with a canine-specific electrochemiluminescent multiplex immunoassay with a measurement range of 2.4-10 000 pg/ml^11^. Six 96 well plates were used in the study and samples were analyzed according to the manufacturer’s instruction, with the exception that the SF samples were diluted 1:2 with diluent provided from the manufacturer prior to analysis. Two control samples based on pooled sera from dogs, with mean IL-6 concentration of 17 pg/ml and 50 pg/ml, were analyzed in duplicate on each plate. The intra- and inter-assay coefficients of variation (CV) were 40.2 and 19.7 % for the low control, and 14.4 and 12.9 % for the high control. Two synovial fluid samples of low and high viscosity, respectively, were spiked with calibrator material to two different concentrations each, and observed recoveries after spiking were 79-102 %. Hyaluronidase treatment of SF samples was tested but did not improve performance (data not shown), and was therefore not used for the study samples. Study samples were analyzed in duplicate in random order, with the exception that serum and SF samples were analyzed on separate plates. Results were excluded if the CV of the duplicate measurements was >30 %, which was subjectively determined to be the maximal acceptable imprecision. Results were also excluded if the sample had signs of iatrogenic bleeding at cytological examination.

### Classification of disease status

#### Suppurative arthritis

Dogs were classified as having suppurative arthritis if the SF NCC was ≥5000 cells/μl with ≥30 % neutrophils, without significant signs of pathological bleeding. If an exact cell count was not available, a moderately to markedly increased cell count on cytological examination was required for classification as suppurative arthritis. Dogs with suppurative arthritis were sub-classified in the following groups: septic arthritis, immune-mediated arthritis, and unclassified cases. Dogs with significant growth of bacteria on SF bacterial culture, and/or intracellular bacteria detected in neutrophils in SF cytology samples, were assigned to the septic arthritis group. To be classified as a case of immune-mediated arthritis, dogs should have negative bacterial culture, no bacteria on SF cytological examination, show clinical improvement within 3 days without being treated with antibiotics and/or repeated joint lavage, and no diagnosis of septic arthritis within 2 weeks after inclusion in the study. Furthermore, the attending clinician should have stated immune-mediated arthritis as the most likely diagnosis in the medical record. Dogs that did not fulfil criteria for septic or immune-mediated arthritis were defined as unclassified cases.

#### Osteoarthritis

Dogs were classified to have OA if the SF NCC count was ≤3000/μl with ≤10 % neutrophils, without significant signs of pathological bleeding. If an exact synovial fluid cell count was not available, the cell count should be low or mildly increased on cytological examination. It was further required that the dog had arthroscopic and/or radiographic evidence of OA described in the medical record.

Dogs that did not fit into the suppurative arthritis or OA group (SF NCC of >3000 but <5000/μl and/or neutrophil numbers >10 % but <30 %), were reported separately.

### Statistical analyses

A statistical software program was used for statistical analyses^12^. Prior to analysis, CRP and IL-6 data were transformed to the base 2-logarithmic scale (log_2_) because of non-normal distributions. Analyte concentrations < LOQ were assigned a value of 0.5 x LOQ for the statistical analyses.

#### Serum C-reactive protein as a discriminative test

Multiple logistic regression analysis adjusted for potential confounders was performed to assess the association between log_2_CRP and outcome, defined as suppurative arthritis/OA. The explanatory variables in the initial model were log_2_CRP concentration, sex, age, body weight and NSAID treatment. Backward stepwise elimination of explanatory variables was used to reduce the model, followed by forward inclusion, until all included variables had a *p*-value <0.05. Explanatory variables, whose inclusion changed the beta coefficient of log_2_CRP by >20 %, were considered possible confounders, and were retained in the model. The goodness of fit was tested with the Hosmer and Lemeshow test. The diagnostic performance of serum CRP for discriminating between suppurative arthritis and OA was assessed by receiver operating characteristic (ROC) curve analysis. Serum CRP was defined as an efficient marker if the lower limit of the 95 % confidence interval of the area under the ROC curve was >0.8. The discriminatory capacity of serum CRP was further evaluated using a decision limit for CRP of 20 mg/l, which was previously established as the clinical decision limit for diagnosing systemic inflammation at the University Animal Hospital, SLU. The decision limit was determined by performing ROC curve analysis of CRP data from dogs with and without known systemic inflammatory disease (data not shown), similarly to what has been previously described [16]. In the current study, correct classification was defined as CRP concentration ≥20 mg/l in a dog with suppurative arthritis, and CRP concentration <20 mg/l in a dog with OA. Comparison of log_2_CRP concentrations in dogs with septic and immune-mediated suppurative arthritis, and in dogs with and without concurrent disease within the suppurative arthritis group, was performed with Student’s *t*-test. Statistical significance was defined as *p* <0.05.

#### Serum C-reactive protein and serum and synovial fluid IL-6 concentrations in dogs with suppurative arthritis, osteoarthritis, and in healthy dogs

Descriptive statistics were presented as median and interquartile range (Q1-Q3) for serum CRP and serum and SF IL-6 concentrations. When SF IL-6 results were available from several joints from the same dog, the mean SF IL-6 concentration was used for calculations. Pairwise comparisons were performed between groups of dogs with suppurative arthritis and OA, and between healthy dogs and dogs with OA respectively, using the unequal variance *t*-test for log_2_CRP and log_2_IL-6 concentrations. Additional comparisons between groups were performed with the Mann–Whitney *U* test for continuous variables, and Fisher’s exact test for categorical data. The *p*-value threshold for statistical significance was adjusted for multiple testing using a Bonferroni correction for 14 tests (0.0036).

#### Correlation between serum and synovial fluid IL-6 and serum C-reactive protein concentrations

The correlations between serum and SF IL-6 concentrations and serum CRP concentrations were evaluated by calculating Spearman rank correlation coefficients. When SF IL-6 results were available from several joints from the same dog, the mean SF IL-6 concentration was used for calculations. Statistical significance was defined as *p* <0.05.

## Results

### Dogs

**Fig. 1:**
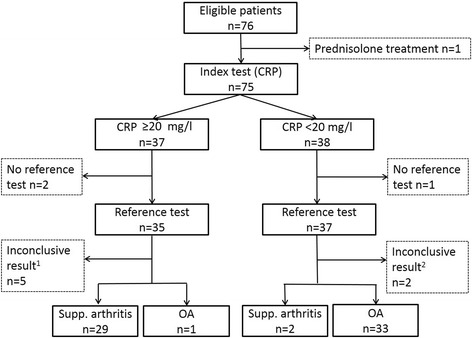
Flow diagram of dogs with joint pain eligible for inclusion in the study. The diagnostic cut-off value for C-reactive protein (CRP) was 20 mg/l. Dogs were classified to have suppurative (supp) arthritis or osteoarthritis (OA) based on synovial fluid analysis (reference test); for classification as OA, signs of OA on radiographs/arthroscopy was further required. Dogs with inconclusive test results were excluded. ^1^Pathological bleeding (*n* = 3) and non-diagnostic sample due to severe iatrogenic bleeding in synovial fluid sample (*n* = 2). ^2^No OA was diagnosed on radiographs/arthroscopy (*n* = 2)

Seventy-six dogs of 29 different breeds were sampled in the study, whereof 11 dogs were excluded (Fig. 1). The remaining dogs were classified as suppurative arthritis (*n* = 31) or OA (*n* = 34) cases (Table 1). For dogs that had multiple joints sampled (*n* = 8), the same classification was obtained regardless of which joint that was evaluated.

**Table 1:** Characterisation of dogs with osteoarthritis (OA), suppurative arthritis and healthy dogs

Parameter	Median (IQR)			*P* values	
	Osteoarthritis (*n* = 34)	Suppurative arthritis (*n* = 31)	Healthy (*n* = 17)	Suppurative arthritis vs OA	Healthy vs OA
Age (years)	2.7 (0.9-6.2)	4.2 (1.6-7.5)	1.8 (1.2-2.7)	0.19	0.33
Weight (kg)	32 (24–39)	28 (19–33)	14 (13–15)	0.11	<0.001
Serum CRP (mg/l)	0.73 (<0.50-2.45)	107 (43–145)	1.26 (0.62-2.32)	<0.001	0.29
Serum IL-6^a^ (pg/ml)	15.0 (10.2-26.5)	101 (47.7-243)	9.2 (8.0-11.4)	<0.001	0.07
Synovial fluid IL-6^b^ (pg/ml)	131 (21.5-450)	19 800 (4030- > 20 000)	<2.4 (<2.4-3.9)	<0.001	<0.001
Sex (male/female)	17/17	18/13	12/5	0.62	0.23
NSAID treatment (treatment/no treatment)	22/12	5/26	0/17	<0.001	<0.001

Serum C-reactive protein (CRP), serum interleukin (IL) 6 and synovial fluid IL-6 concentrations in dogs with osteoarthritis (OA), suppurative arthritis, and in healthy dogs. Data is presented as median and interquartile range (IQR). Pairwise comparisons were performed between groups of dogs with OA and suppurative arthritis, and between dogs with OA and healthy dogs. NSAID; non-steroidal anti-inflammatory drug

^a^Serum IL-6 results available from dogs with OA (*n* = 29), suppurative arthritis (*n* = 29), and healthy dogs (*n* = 7)

^b^Synovial fluid IL-6 results available from dogs with OA (*n* = 19), suppurative arthritis (*n* = 29), and from healthy dogs (*n* = 14)

In dogs with suppurative arthritis the NCC was moderately to markedly increased on cytological examination, and the percentage of neutrophils was 65–96 %. Cell counts performed in a hemacytometer (*n* = 22) were between 6000–272 000/μl. Intracellular bacteria were present on cytological examination in the sample of one dog that also had a positive bacterial culture. Ragocytes or lupus erythematosus cells were not detected in any of the dogs. Bacterial cultures were performed in blood culture containers in 13 samples, and from sterile cotton swabs in 18 samples. Bacterial culture results were positive in 4 dogs that were subsequently classified as septic arthritis cases, and negative in the other dogs. Immune-mediated arthritis was diagnosed in 8 dogs. The remaining 19 dogs with suppurative arthritis were treated with antibiotics but infection was not confirmed, and therefore they were allocated to the group of unclassified suppurative arthritis cases. Concurrent disease was recorded in 13/31 dogs with suppurative arthritis, most commonly gastrointestinal disease (*n* = 7). Laboratory test abnormalities included non-regenerative anemia (*n* = 6) neutrophilia (*n* = 12), left shift with band neutrophils >0.5 × 10^9^/L (*n* = 13), lymphopenia (*n* = 15), thrombocytopenia (*n* = 5), hypoalbuminemia (*n* = 13), and increased alanine aminotransferase (*n* = 2) and alkaline phosphatase (*n* = 2) activity. Nineteen dogs had neutrophilia and/or left shift, whereas neutrophil numbers and morphology were assessed to be normal in the remaining 12 dogs. In one dog, inclusions consistent with *Anaplasma phagocytophilum* were present in blood neutrophils.

Dogs with OA had osteochondritis dissecans (*n* = 10), cranial cruciate ligament injury (*n* = 12), unspecified cartilage defect (*n* = 7), fragmented coronoid process (*n* = 2) and/or synovitis (*n* = 10). Synovial fluid NCC were 150–1700 /μl with 0–10 % neutrophils, and there were no abnormal morphological findings on cytological examination. Bacterial cultures were performed on cotton swabs (*n* = 26) and in blood culture containers (*n* = 8), and were negative in all dogs. No dog had concurrent disease reported. Abnormalities on hematology and biochemistry testing included non-regenerative anemia (*n* = 5), neutropenia (*n* = 5), lymphopenia (*n* = 6) and hypoalbuminemia (*n* = 8).

Antibodies against *Anaplasma phagocytophilum*, *Borrelia burgdorferi*, or both, in a titre ≥80, were present in 16/31 (52 %) dogs with suppurative arthritis and in 13/34 (38 %) dogs with OA; there was no significant difference between the groups (*p* = 0.22).

Of the 18 dogs sampled as a comparative group, one was excluded because of moderately increased ALAT activity. Synovial fluid NCC in the remaining dogs were 160-810/μl, and no neutrophils were present on cytological examination.

### Serum C-reactive protein as a discriminative test

In the logistic regression analysis with suppurative arthritis/OA as outcome, the final model included log_2_CRP (*p* = 0.03, OR 7.37) and sex (*p* = 0.12, OR 0.005) as explanatory variables. The interpretation of OR for log_2_CRP was that for a doubling of serum CRP concentration, the odds of having suppurative arthritis increased by a factor of 7.37. The area under the ROC curve (95 % CI) was 0.99 (0.97-1.00). Based on ROC curve analysis, the optimal decision limit for serum CRP for discriminating between dogs with suppurative arthritis and OA was 3.5 mg/l which was lower than the locally established clinical decision limit, 20 mg/l. Using the latter decision limit, two dogs in the suppurative arthritis group with serum CRP concentrations of 6.0 and 3.9 mg/l were misclassified, as well as one dog in the OA group with serum CRP concentration of 68 mg/l (Fig. 1).

In dogs with septic suppurative arthritis (*n* = 4), the median (range) serum CRP concentration was 81.5 (6.0-189) mg/l. The corresponding serum CRP concentrations in dogs with immune-mediated suppurative arthritis (*n* = 8) was 118.0 (31–229) mg/l. Serum CRP concentrations did not differ significantly between dogs with septic and immune-mediated suppurative arthritis (*p* = 0.46). There was no significant difference in CRP concentration between dogs with and without concurrent disease within the suppurative arthritis group (*p* = 0.25).

### Serum C-reactive protein and serum and synovial fluid IL-6 concentrations in dogs with suppurative arthritis, osteoarthritis, and in healthy dogs

Results from serum CRP measurements were available from all dogs (*n* = 82). Interleukin-6 results were excluded from 17 serum and 11 SF samples, because the CV of duplicate measurement was >30 %. Synovial fluid IL-6 results were further excluded because of mild iatrogenic blood contamination (*n* = 9). Dogs with suppurative arthritis had increased serum CRP and serum and SF IL-6 concentrations compared to dogs with OA (Table 1). Dogs with OA had higher SF IL-6 concentrations compared to healthy dogs, but there was no difference between these two groups for serum IL-6 or serum CRP (Table 1).

### Correlation between serum and synovial fluid IL-6 and serum C-reactive protein concentrations

There was a positive correlation between SF IL-6 and serum CRP concentrations (r_s_ = 0.733, *p* < 0.001), and between serum IL-6 and serum CRP concentrations (r_s_ = 0.729, *p* < 0.001).

## Discussion

The results from the current study showed that serum CRP concentrations discriminated well between dogs with suppurative arthritis and OA, with 62 out of 65 dogs (95 %) being correctly classified using a pre-existing clinical decision limit for CRP. It has been argued that a complete physical examination is usually sufficient to adequately distinguish a dog with suppurative arthritis from a dog with OA [15], but clinical signs may be unspecific [8, 10, 11] and physical examination is subjective in contrast to CRP, which is an objective marker and thus less biased by level of clinical expertise. The logistic regression model confirmed that increased serum CRP concentrations were associated with a diagnosis of suppurative arthritis, compared to a diagnosis of OA. Although sex remained as an explanatory variable in the final model, we do not consider this to be of clinical importance. Concurrent disease was not included as an explanatory variable in the logistic regression model, because all dogs with concurrent disease belonged to the suppurative arthrithis group which would yield inadequate estimations. Instead, it was tested whether there was a difference in CRP concentration in dogs with and without concurrent disease within the suppurative arthritis group. No such difference was found, and it was concluded that the increased CRP concentration found in dogs with suppurative arthritis was not due to presence of concurrent disease only.

There were two dogs in this study that had suppurative arthritis based on SF examination, but yet low serum CRP concentrations. This is not optimal, considering that suppurative arthritis should be promptly treated [9] and that a false negative CRP result may delay correct diagnosis if used as sole means of differentiation. Both dogs lacked other clinical and laboratory signs indicative of systemic inflammation (data not shown), and it is possible that they had purely local joint inflammation that did not elicit a systemic inflammatory response.

A definitive diagnosis of septic or immune-mediated arthritis was not possible to establish in all dogs, and one reason for this was that many dogs with suppurative arthritis received antibiotics despite negative bacterial culture. Frequent antibiotic treatment has previously been reported in dogs with immune-mediated suppurative polyarthritis [11], which can be explained by the difficulty in excluding infection in these patients. Furthermore, underdiagnosis of septic arthritis in the current study was possible considering that the more sensitive method of bacterial cultures in blood culture containers was not possible to perform in many cases because of insufficient SF volumes [12]. Due to the low number of animals with confirmed septic or immune-mediated suppurative arthritis, the power of detecting an existing difference in serum CRP concentration between groups was low. However, considering the marked overlap in serum CRP concentrations in dogs with septic and immune-mediated arthritis, it is unlikely that serum CRP would be an efficient discriminatory test for these two conditions. This is in line with the results of previous studies, in which serum CRP concentrations did not differ between dogs with septic and non-septic inflammatory disease [23, 30, 31]. Although specific situations may exist where serum CRP indeed could be helpful for predicting presence of bacterial infection, as suggested in dogs with respiratory disease [32], this is not generally applicable.

Dogs with suppurative arthritis had increased serum CRP and serum IL-6 concentrations compared to dogs with OA, in agreement with what has been previously described [15]. Synovial fluid IL-6 concentrations were markedly increased in the suppurative arthritis group compared to the OA group, which is in accordance with the higher SF IL-6 activity earlier found in dogs with rheumatoid suppurative arthritis compared to dogs with OA [33]. However, in another study no difference in SF IL-6 mRNA expression was detected when comparing dogs with immune-mediated arthritis and OA [34]. Different methodologies used for measuring IL-6 could possibly explain the diverging results in different studies.

Dogs with OA were compared to healthy dogs in order to investigate if there was indication of local inflammation in joints affected by OA, as has been previously described [4, 35]. The finding of increased SF IL-6 concentrations in dogs with OA compared to healthy dogs, without increased serum CRP concentrations, supported that a local inflammation without significant systemic effects was present. However, results from the comparisons between healthy dogs and dogs with OA in the current study need to be interpreted with caution considering that the comparative group was not representative of all dogs with OA. Factors such as breed and age may affect serum CRP concentrations [36, 37], and it is desirable to control for these possible confounders when investigating CRP concentrations in the low range. This was not possible in the current study because all healthy dogs were Beagle dogs of similar age. The frequency of NSAID treatment was also significantly different between groups and because NSAID treatment may influence IL-6 concentrations [38, 39], secondary effects on CRP concentration could possibly occur. However, treatment with NSAID did not significantly affect serum CRP concentrations in three previous studies [18–20], and it was therefore considered less likely that NSAID treatment biased CRP results in the current study.

There was a positive correlation between IL-6 and CRP concentrations which was expected, as IL-6 induces hepatic CRP production [13, 14]. A full validation of the IL-6 assay was not performed, which should be taken into consideration when interpreting IL-6 results. Suggested validation protocols for cytokine immunoassays have been published [40], but such studies are demanding to carry out and often lacking in veterinary medicine. In the current study, only minor validation experiments were performed (data not shown). However, the assay did detect the expected increased IL-6 concentrations in dogs with suppurative arthritis. This indirectly implied that it was a useful assay, not affected by the suspected low analytical sensitivity that has been described for another canine IL-6 immunoassay [41]. For diagnosing suppurative arthritis in a clinical setting measurement of IL-6 would not be the test of choice though, because CRP was superior over IL-6 for discriminating between dogs with OA and dogs with suppurative arthritis (data not shown). Further advantages of the CRP assay include that it is thoroughly validated, fully automated and more cost effective compared to the IL-6 assay. C-reactive protein also has the advantage of being more stable during storage than IL-6 [42, 43]. It has previously been reported that IL-6 is stable for approximately 2 years of freeze storage [42], and for this reason the maximal storage time in the current study was set to 2 years.

There was no difference in the number of dogs that were seropositive for *Anaplasma phagocytophilum* and *Borrelia burgdorferi* in dogs with suppurative arthritis and OA. Although arthritis has been described in dogs experimentally infected with *Borrelia burgdorferi* [44, 45], and infection with *Anaplasma phagocytophilum* has been suspected to cause immune-mediated polyarthritis [22], it is often difficult to establish causality between infection and arthritis in a clinical setting which is why determination of a single antibody titre is considered to be of limited diagnostic value. All healthy dogs in this study were seronegative, which could be explained by the fact that they were purpose bred research dogs and most of them had not been exposed to ticks.

The clinical decision limit of serum CRP for diagnosing systemic inflammation was set to 20 mg/l, which is the limit used at the University Animal Hospital where the study was conducted. This limit is comparable to a previously recommended clinical decision limit for serum CRP of 16.8 mg/l [16], established with a CRP method giving similar results as the assay used in the current study [28]. The optimal decision limit for serum CRP based on ROC curve analysis in the present study was 3.5 mg/l, but in a clinical setting such low decision limit would likely give rise to an unacceptably large number of false positive results. Further studies in an independent setting would be needed to quantify the specificity of such a low cut-off.

One important limitation of the current study was that only dogs admitted for arthrocentesis or other invasive procedure in the joint were eligible for inclusion. This approach was chosen in order to avoid arthrocentesis being performed for study purposes solely, but the consequence was that the sampling population was not representative of the patient population where CRP testing would probably be most useful, i.e. in the early diagnostic workup of dogs with joint pain of unknown etiology. None of the dogs with OA was reported to have concurrent disease, probably because dogs with ongoing systemic inflammation would normally not be referred to undergo a surgical procedure to diagnose or treat OA. In a clinical setting it may be more likely to encounter dogs suffering from both OA and concurrent inflammatory disease, thus decreasing the efficacy of serum CRP for discriminating between suppurative arthritis and OA. Despite this, considering the pronounced difference in CRP concentration between dogs with suppurative arthritis and OA, it is concluded that CRP is a promising diagnostic marker in these patients. Future studies should include prospective evaluation of dogs with joint pain admitted to primary care clinics, in order to evaluate if CRP measurements will improve patient care in a cost-effective way.

## Conclusions

Serum CRP concentration discriminated well between dogs with suppurative arthritis and OA. There was no difference in CRP concentration between dogs with septic and immune-mediated arthritis, but only few dogs were included in this comparison and therefore the power of detecting an existing difference in serum CRP concentration between these two groups was low. Concentrations of serum CRP and serum and SF IL-6 were higher in dogs with suppurative arthritis compared to dogs with OA.

## Abbreviations

CRP: C-reactive protein
CV: Coefficient of variation
IL: Interleukin
LOQ: Limit of quantification
NSAID: Non-steroidal anti-inflammatory drugs
NCC: Nucleated cell count
OA: Osteoarthritis
ROC: Receiver operator characteristic
SF: Synovial fluid
WBC: White blood cell

## Footnotes

1 Vacutainer, Becton-Dickinson, Stockholm, Sweden

2 Micro tube, Sarstedt AG & Co, Numbrecht, Germany

3 Abbott Architect c4000, Abbott Park, IL, USA

4 Advia 2120, Siemens Healthcare Diagnostics, Deerfield, IL, USA

5 cMedonic CA 620/530 Vet Analyzer, Boule Medical AB, Stockholm, Sweden

6 Sigma-Aldrich, St Louis, MO, USA

7 BOF, Substratlab, National Veterinary Institute, Uppsala, Sweden

8 Culturette, Becton-Dickinson, Stockholm, Sweden

9 Gentian cCRP, Gentian AS, Moss, Norway

10 Gentian hsCRP, Gentian AS, Moss, Norway

11 Canine Pro-inflammatory Panel 3 Assay, Ultra-sensitive kit, Meso Scale Discovery, Maryland, USA

12 SAS 9.3, SAS Institute Inc., Cary, NC, USA
